# Ileocolic intussusception secondary to Peutz-Jeghers polyp: the need for oncological resection surgery

**DOI:** 10.1093/jscr/rjae489

**Published:** 2024-08-08

**Authors:** Kristali Ylli, Wala Eljack, Brian Hayes, Thomas Murphy

**Affiliations:** Department of Surgery, Mercy University Hospital, Grenville Place, Cork T12 WE28, Ireland; Department of Surgery, Mercy University Hospital, Grenville Place, Cork T12 WE28, Ireland; Department of Histopathology, Cork University Hospital, Wilton, Cork T12 DC4A, Ireland; Department of Surgery, Mercy University Hospital, Grenville Place, Cork T12 WE28, Ireland

**Keywords:** adult intussusception, oncological resection, Peutz-Jeghers polyp, malignancy

## Abstract

In this case report, we detail the management of a woman in her late 30s with ileocolic intussusception, emphasizing the high malignancy risk inherent in adult intussusception cases. Given the patient’s acute symptoms and significant family history of ovarian and breast cancers, radical oncological resection was pursued. The surgical intervention comprised a right hemicolectomy and right ovarian cystectomy, with histopathological findings revealing a Peutz-Jeghers polyp and benign thyroid tissue, but no malignancy. This case underscores the imperative for a surgical approach that anticipates the potential for malignancy in adult intussusception, advocating for radical resection as a fundamental strategy, even in the absence of confirmed malignant histopathology, to ensure comprehensive management and alignment with oncological best practices.

## Introduction

In adults, intussusception is an atypical and complex clinical presentation, characterized by the invagination of a segment of the intestine into an adjoining segment [1]. Unlike in children, where intussusception is more commonly idiopathic, the occurrence in adults frequently heralds underlying pathologies, with malignancies accounting for a significant proportion of cases [2]. The differential diagnosis for adult intussusception is broad, yet neoplastic processes are often implicated as causative factors, necessitating an aggressive diagnostic and therapeutic approach [2]. The presence of a neoplasm as a lead point in adult intussusception underscores the critical importance of surgical intervention, not merely to alleviate the physical obstruction but also to address the oncological underpinnings of the condition [3].

In managing adult intussusception, the strategy extends beyond mere mechanical repair, highlighting the necessity for a detailed surgical plan that integrates oncological standards. This paradigm ensures not only the resolution of the immediate intestinal obstruction but also the thorough assessment and removal of potential malignancies, aiming to prevent further disease progression [4]. This case report exemplifies the essential integration of oncological vigilance and surgical expertise in managing a rare case of adult intussusception highlighting the critical importance of surgical resection in such contexts.

## Case report

We report a complex case of a woman in her late 30s who presented to the emergency department with severe, nonlocalized lower abdominal pain radiating to the back, accompanied by recurrent vomiting and diarrhoea. The pain intensity was rated at 9/10, underscoring the severity of her condition. Physical examination revealed notable tenderness and guarding in the suprapubic and right iliac fossa areas, with pronounced high-pitched bowel sounds, suggesting acute abdominal pathology.

**Figure 1:**
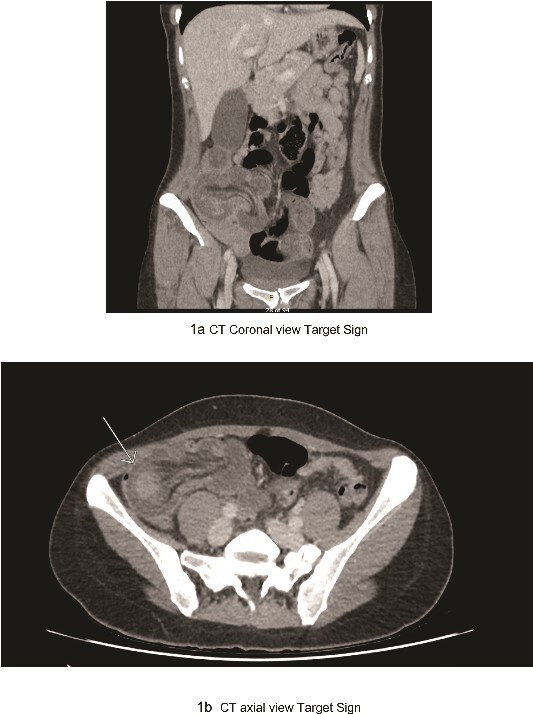
(a) CT coronal view target sign; (b) CT axial view target sign.

The patient’s significant familial history of ovarian and breast cancers immediately prompted considerations of underlying malignancy. Initial laboratory tests indicated leucocytosis and elevated inflammatory markers, while radiologic assessment through abdominal and pelvic computed tomography scans revealed ileocolic intussusception (Fig. 1a and b) and an ovarian teratoma, raising immediate concerns for a malignant aetiology given the rare presentation in an adult.

In light of the high risk of malignancy associated with adult intussusception—reported as up to 50% in aetiological studies—an exploratory laparotomy was deemed essential. The surgery confirmed the diagnosis of ileocolic intussusception and identified a mature cystic teratoma on the right ovary. The surgery was meticulously planned and executed with the intent of oncological resection. An ileocolic intussusception was identified, and the colon was congested and partially ischaemic, necessitating an extended resection due to both oncological considerations and the necrosis of the colon. There was no evidence of metastatic disease within the peritoneal cavity.

A right ovarian cyst was identified with no particular concerning features. The right colon was mobilized in the standard fashion. The right ureter, gonadal vessels, and duodenum were identified. A right hemicolectomy was performed with a functional end-to-end anastomosis fashioned with a gastrointestinal anastomosis stapler (GIA) 55 and a TX60. A right ovarian cystectomy was performed with preservation of the right ovary. On the fifth day following the right hemicolectomy and right ovarian cystectomy, the patient was allowed to go home with a straightforward postoperative course and no complications.

**Figure 2:**
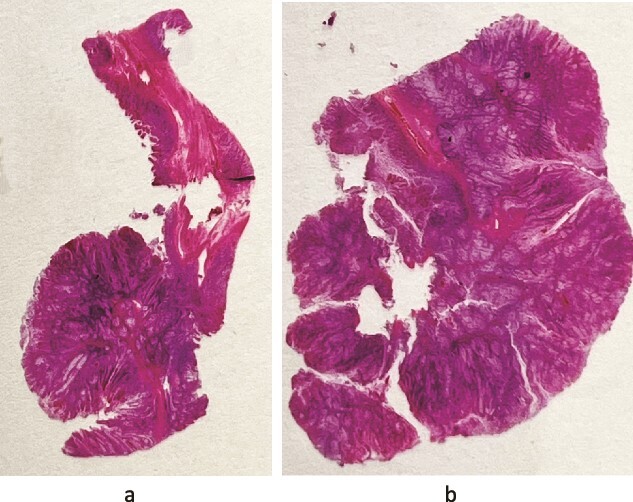
(a and b) Photography of the glass slide: they give an impression of the architecture of these hamartomatous polyps—nests of nondysplastic glands separated by arborizing strands of smooth muscle.

**Figure 3:**
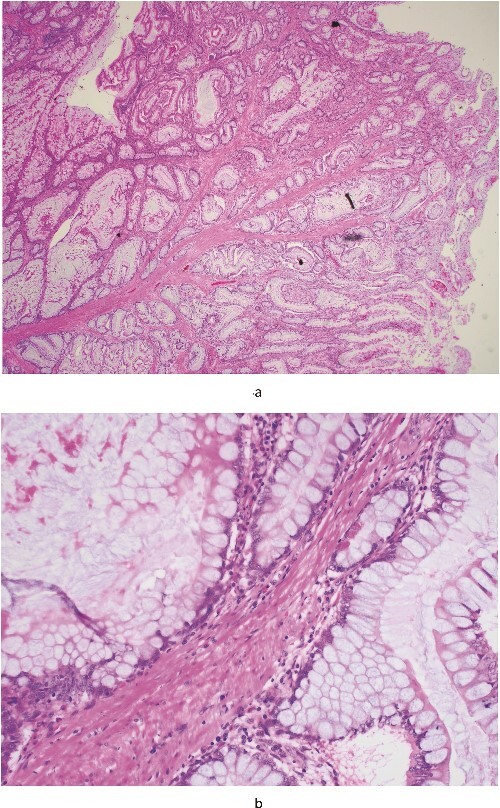
(a) (20×) is a low-power microscopic image of the polyp; the eosinophilic (pink!) strands of tissue running from the bottom left towards the centre, where they progressively divide, are the smooth muscle fibres and (b) (400×) is a high-power view of the epithelial cells—they have small round basal nuclei and abundant mucinous apical cytoplasm, and are nondysplastic.

Histological analysis post-surgery revealed mucosal necrosis, submucosal oedema, and vascular congestion, aligning with the anticipated diagnosis of intussusception. A 3.5-cm Peutz-Jeghers polyp (Figs 2 and 3), situated at the lead point of the intussusception, and a 7.5-cm benign ovarian teratoma featuring prominent thyroid tissue were identified, with no signs of dysplasia or malignancy detected. This outcome highlights the efficacy of conducting surgery with an oncological resection perspective, especially considering the high malignancy risk in adult intussusception cases.

A multi-disciplinary team discussion was held regarding this case and genetic testing for STK11/LKB1 gene to exclude Peutz-Jeghers syndrome was recommended. STK11 testing that is for Peutz-Jeghers syndrome was negative. This outcome not only informed the post-operative management strategy but also provided valuable insight into the patient’s genetic profile, reinforcing the importance of a comprehensive approach that integrates genetic testing into the post-surgical care protocol for cases with an elevated risk of hereditary conditions.

## Discussion

This case report illustrates a significant clinical scenario where a patient with adult intussusception underwent surgical intervention with the underlying premise of addressing a potential malignancy. Adult intussusception is a relatively rare clinical entity, markedly different from its paediatric counterpart, primarily due to the high incidence of associated malignancies [5]. Studies indicate that neoplastic causes account for up to 50% of adult intussusception cases, in sharp contrast to paediatric instances, which are predominantly idiopathic [6]. Various case reports and systematic reviews have advocated for oncological resection due to the notable risk of malignancy. For instance, the systematic review and meta-analysis by Hong *et al*. [7], titled ‘Adult intussusception: a systematic review and meta-analysis’, concludes that surgery is fundamental in managing adult intussusception. It highlights that enteric intussusception, being the most prevalent form in adults, should typically be approached with initial reduction and subsequent resection, given that metastatic carcinoma, lymphoma, and gastrointestinal stromal tumour are frequently the underlying malignant causes. This significant statistic emphasizes the critical need for an oncological perspective in the surgical management of adult intussusception [6].

The decision for an exploratory laparotomy, followed by a right hemicolectomy and ovarian cystectomy in this case, was driven by the dual objectives of resolving the mechanical obstruction and excising potential malignancy. The surgical strategy was meticulously planned, incorporating oncological principles from the outset, given the significant likelihood of encountering malignancy as the underlying aetiology. Additionally, the decision to extend the resection was influenced by the presence of colon necrosis. This approach is pivotal, not only for the immediate resolution of the presenting symptoms but also for the long-term prognosis of the patient [8].

Histopathological examination in this instance revealed a Peutz-Jeghers polyp and a benign ovarian teratoma, with no evidence of malignancy. While the immediate postoperative findings may not have confirmed a malignant cause, the clinical rationale for an oncologically focused surgical approach remains unchallenged. This is especially relevant in the context of the patient’s family history of cancer, which adds a layer of complexity to the case, emphasizing the importance of considering genetic predispositions to malignancy [9].

Furthermore, the negative result from the genetic testing for Peutz-Jeghers syndrome, while providing relief, does not diminish the necessity for vigilance and proactive management strategies in similar cases [10]. The genetic aspect of this case highlights the evolving understanding of the interplay between genetic factors and surgical decision-making, particularly in cases with a high index of suspicion for malignancy [11].

## Conclusion

In conclusion, this case reinforces the indispensable role of oncological resection in the management of adult intussusception, guided by the principle of erring on the side of caution in the face of potential malignancy. It accentuates the importance of a multidisciplinary approach, integrating surgical, histopathological, and genetic insights to navigate the complexities of such cases effectively [12]. Our management approach and conclusions are in alignment with the prevailing studies in the literature, reinforcing the established protocols for addressing this condition.

As surgical practices continue to evolve, the integration of oncological principles in the management of gastrointestinal pathologies underscores a paradigm shift towards more holistic and patient-centric care [13].
